# Comparison of two different ways to apply a circular plaster cast for distal radius fractures: biomechanical study

**DOI:** 10.1186/s13018-021-02256-1

**Published:** 2021-01-30

**Authors:** Alejandro Espejo-Reina, María T. Carrascal-Morillo, Alberto D. Delgado-Martínez

**Affiliations:** 1 Deparment of Orthopaedic Surgery, Clínica Espejo, Paseo Reding, Málaga, Spain; 2 Deparment of Orthopaedic Surgery, Hospital Vithas Parque San Antonio, Málaga, Spain; 3 Higher Technical School of Industrial Engineering, Madrid, Spain; 4 Department of Orthopaedic Surgery, Complejo Hospitalario de Jaén, Jaén, Spain; 5 Department of Surgery, University of Jaén, Jaén, Spain

**Keywords:** Wrist fracture, Plaster of Paris, Plaster cast, Conservative treatment, Splint, Immobilization, Distal radius fracture

## Abstract

**Introduction:**

Although conservative treatment with circular plaster cast is the most commonly used method in distal radius fractures, the best method to apply it remains unclear.

**Material and methods:**

Two frequently used configurations of circular plaster cast (with and without a splint) were selected to compare. Group C was applied only with circular bandages (three units) and group S with a splint (one unit) and over it, a circular bandage (two units). Both configurations had the same weight. Five prototypes of each group were built and mechanically tested. Three-point flexural tensile strength and maximum deflection were measured and compared.

**Results:**

The previously splinted prototypes (group S) obtained higher tensile strength with the same weight (*p* < 0.05).

**Discussion:**

No other study regarding strength and configuration of circular casts for distal radius fractures immobilization has been previously published, leading to a high variability in construction among orthopedic surgeons. Data confirms that applying a splint before circular bandage offers more mechanical resistance to the cast in flexion, with the same weight.

**Conclusion:**

Applying a splint before circular bandage for plaster casts used for distal radius fractures make them more resistant to usual forces.

## Introduction

Distal radius is the location for one-sixth of the whole body fractures. Ninety percent of them are extraarticular, known as Colles’ fractures [1].

Conservative treatment, namely immobilization without surgery, is the most commonly applied treatment in this type of fractures. Several studies suggest that this treatment achieves similar clinical results to surgery in patients older than 60 years [2–6], emphasizing the high number of patients treated conservatively in common practice.

Although some new materials have been developed [7–13], plaster cast remains the gold standard and the most frequently used form of immobilization [2, 14]. Plaster as a material is isotropic. Thus, it is able to resist forces in any direction with the same strength. Nevertheless, the plaster cast has a form (shape) that distributes the material in a special form to get more resistant in some directions, so it is anisotropic.

There are different forms of applying the cast for this type of fractures [15–17], but there is no conclusive data to indicate that one method (long versus short plaster casts [15], double thickness against reinforced single thickness [16], single reinforcing ridges [17]) is superior to other. Our current practice is to apply a circumferential closed molded cast directly onto the arm (when reduction is not necessary). If reduction is necessary or inflammation is expected, we apply an open circumferential cast that is changed to a circumferential closed molded cast after inflammation has subsided (about 7–10 days later).

Usual practice is to maintain the cast for 5–6 weeks. Recent reports indicate that immobilization cannot be shortened without losing reduction [18]. So getting a strong construction of the cast is important, with the minimum weight possible, so the cast does not deteriorate with time.

Classical texts indicate two basic ways to apply a circumferential antebraquial cast [19]. Italian school, popularized by Morandi, consisted of applying circumferential plaster cast bandages directly over padding. German school, popularized by Böhler, apply very little (or no) padding, then a dorsal plaster splint, and over it, the rest of the circumferential plaster cast bandages [19]. Final result in terms of contention of fracture seems similar, but strength to usual forces in 6 weeks is unknown.

The aim of this work is to compare the resistance to usual forces (flexion) of two commonly used configurations of cylindrical plaster cast: one applying a splint before circumferential casting, and the other with circumferential casting alone, using the same quantity of plaster (same weight) in both configurations.

## Material and methods

**Fig. 1:**
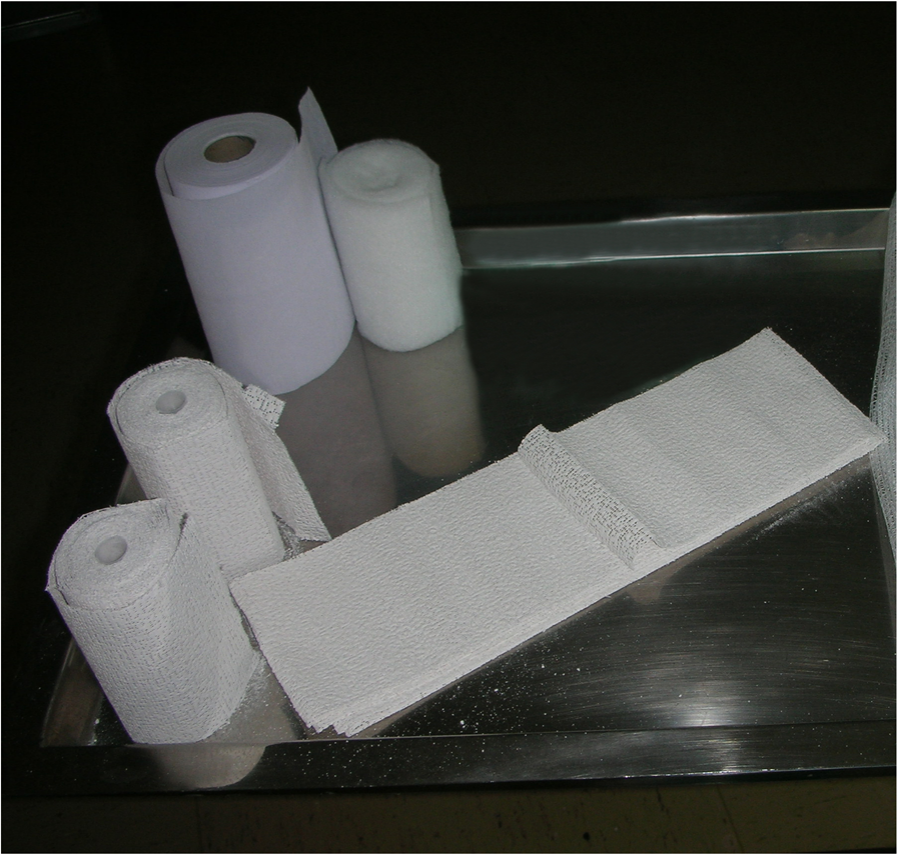
Material used for the plaster cast preparation. A layer of standard padded bandage (Texban-s®, Texpol, 100% polyester, 10 × 270 cm) and three plaster bandages (Guypse®, BSN medical, 10 × 270 cm) (one of them has been flattened into a splint for group S). In both prototypes, the quantity of plaster of Paris (weight) is the same

Using a specifically modified mannequin’s forearm as a human model, ten plaster cast prototypes were made, based in two techniques of cylindrical plaster cast application for the treatment of distal radius fractures (five prototypes with each technique): Same quantity of material (Cast) was used for both techniques (Fig. 1). When the circumferential bandage was applied, it was applied uniformly, continuously, without reinforcement at any point.

**Fig. 2:**
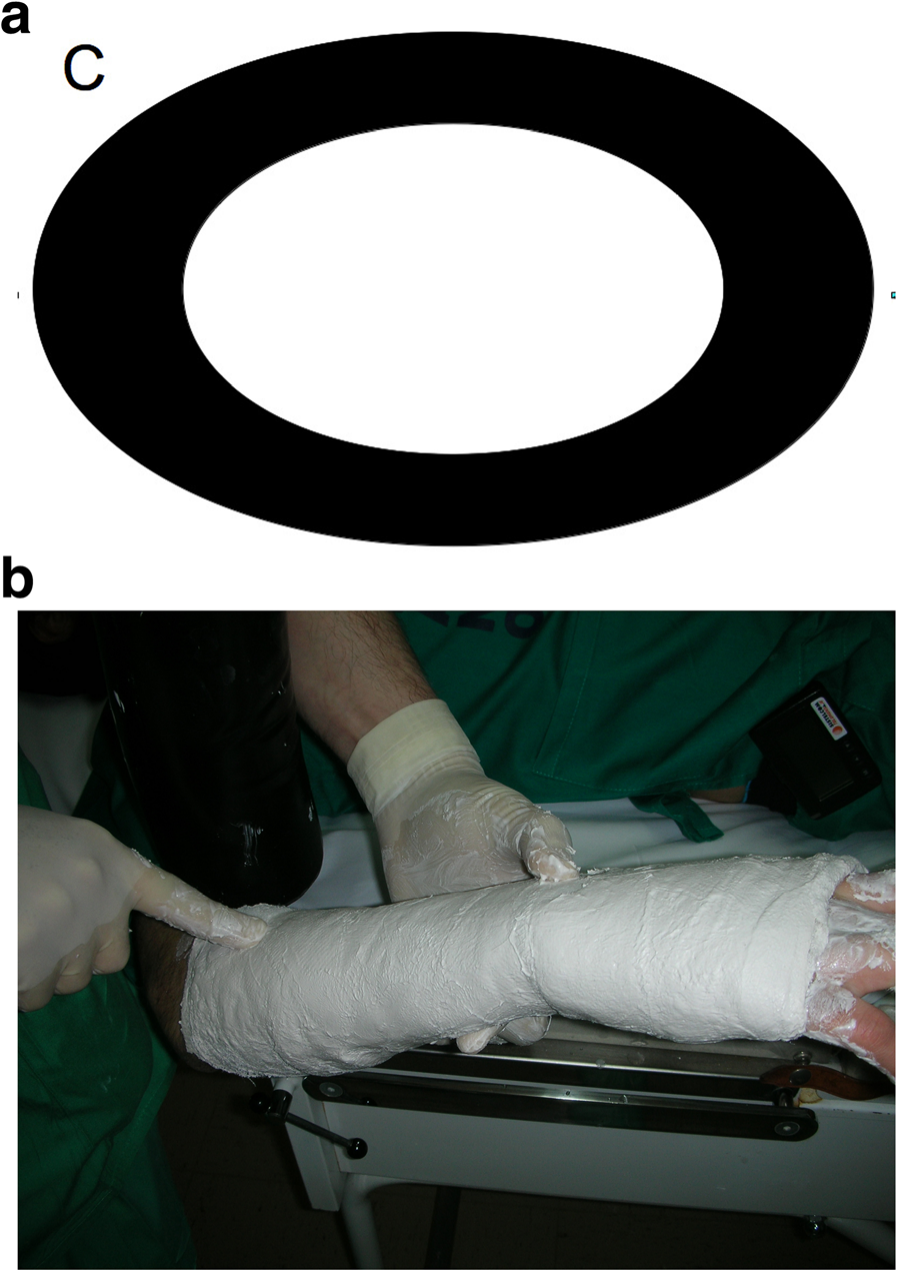
Circular plaster alone. **a** Diagram of the system of applying the cast (transversal cut). **b** Application in clinical setting

**Fig. 3:**
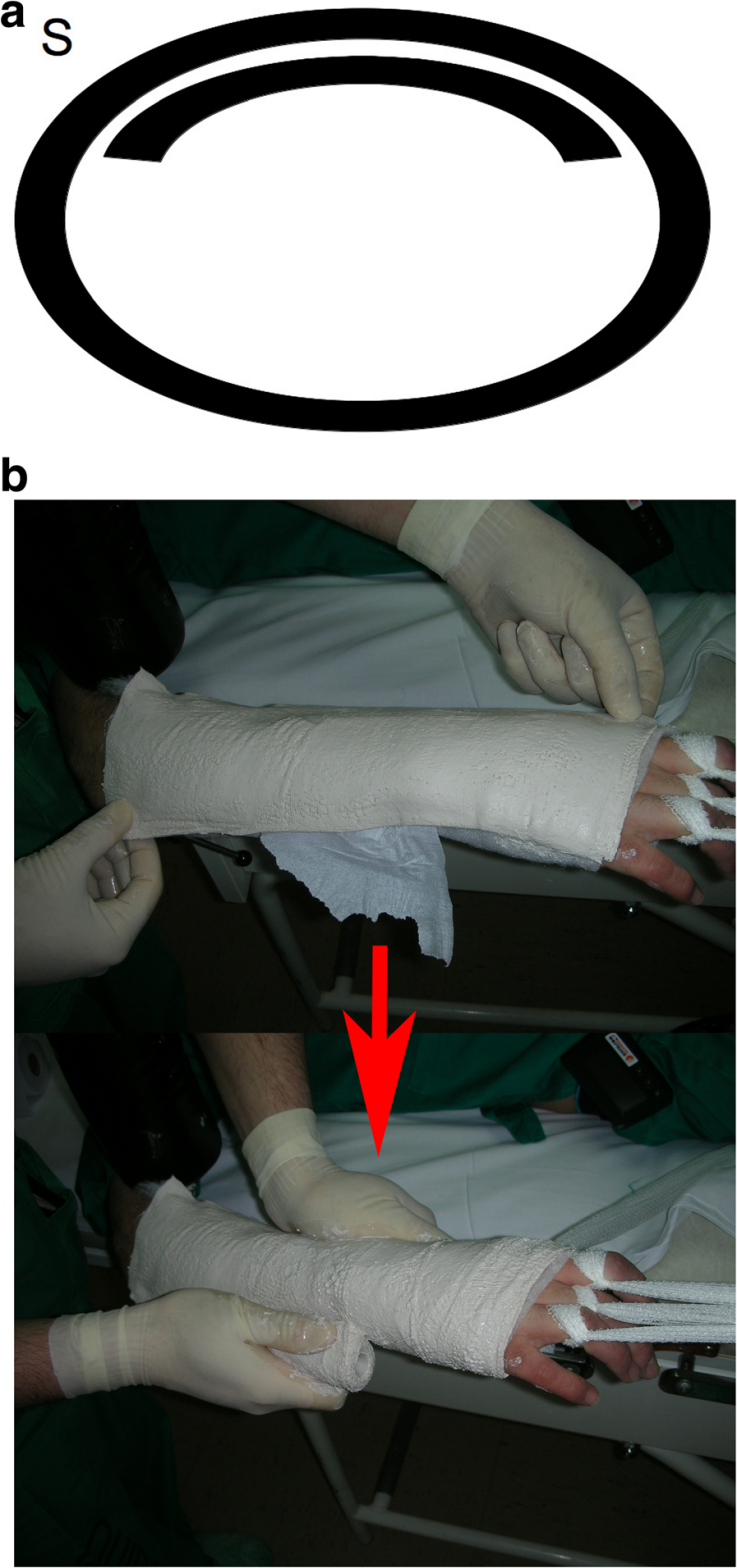
Splint applied under the circular plaster cast (before it). **a** Diagram of the system of applying the cast (transversal cut). **b** Application in clinical setting

- Circumferential casting alone (C): A layer of standard padded bandage (Texban-s®, Texpol, 100% polyester, 10 × 270 cm) was applied. Over it, three plaster bandages (Guypse®, BSN medical, 10 × 270 cm) were applied circumferentially, on a regular way, molded on the mannequin’s forearm*.* Each prototype was denominated with the letter C and a number, according to the order in which they were included in our study (e.g., C1.) (Fig. 2a, b).
- Circumferential casting with a splint (S): A layer of standard padded bandage was applied, similar to that applied to group C. Over it, one of the plaster bandages was applied as a dorsal splint, and then, the other two plaster bandages were placed on a circumferential way, molded on the splint, and then on the mannequin's forearm. Each prototype was denominated with the letter S and a number, according to the order in which they were included in our study (e.g., S1.) (Fig. 3a, b).

**Fig. 4:**
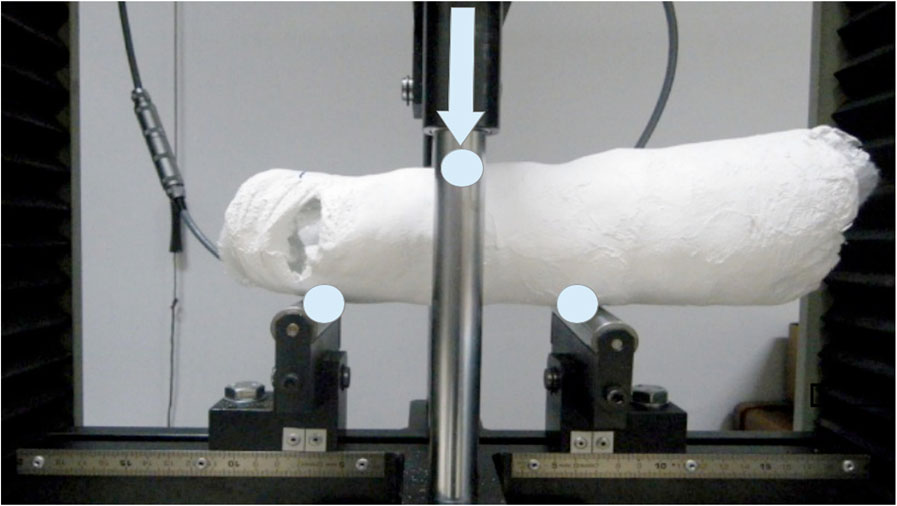
Specimen installed on machine for testing. A load is applied in the central bar to get a three-point bending test, in a similar way as they are loaded in the patient. The circles show the equidistant deflection points (separated by 7 cm) and the arrow shows the direction of the single load applied

After 2 days (time necessary for the plaster to set completely), the mannequin’s forearm was withdraw (mannequin was detachable in pieces as not to damage the cast). The prototypes underwent a three-point bending mechanical test in flexion. A single load was given midway between both ends of the prototype using an electromechanical press machine manufactured by Schenk-Trebel (Fig. 4). Data obtained were tensile strength in Newtons and maximum deflection in millimeters before breakage.

Results were analyzed between groups using the free software “R-commander” (R Development Core Team (2011) http://www.R-project.org/). Variables were described in general and specifically for each kind of plaster cast. These variables were mean, median, standard deviation, and range (minimum and maximum). Nonparametric tests (Mann-Whitney test for independent samples, with a degree of significance of *α* < 0.05) were applied for every variable in the contrast of hypothesis that was as follows:

- *Null hypothesis (H0)*. The variable has no statistically significant differences according to the type of plaster cast.
- *Alternative hypothesis (H1)*. The variable has statistically significant differences according to the type of plaster cast.

## Results

**Fig. 5:**
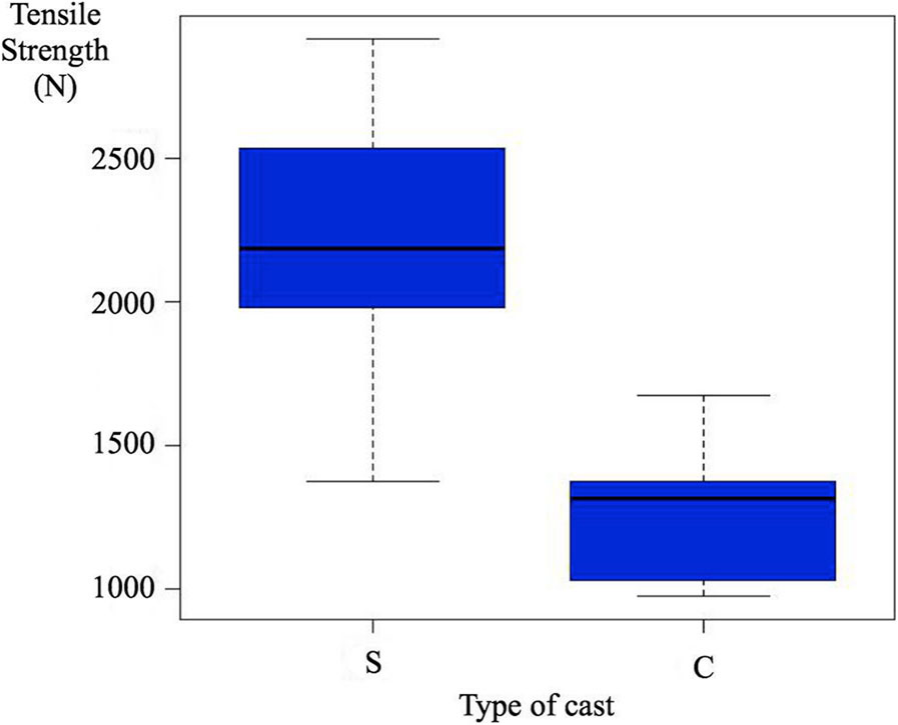
Box plots showing the maximum load (tensile strength) before breaking in each group. S = circumferential casting with a splint group, and C = circumferential casting alone group. The area within the box includes values between the 25 percentile and the 75 percentile, while the black line represents the median. See that strength is superior in the circumferential casting with a splint group (S)

Data obtained from mechanical tests are summarized in Table 1 and Fig. 5. The mean tensile strength in circumferential casting with a splint (S) group was higher than in circumferential casting alone (C) group (nearly double, 2195 N versus 1273 N), with a statistically significant difference (*p* = 0.021). Mean maximum deflection (*p* = 0.009) was also superior for the circumferential casting with a splint group (S), with statistically significant differences (Table 1).

**Table 1:** Results of biomechanical testing and statistical analysis of biomechanical data

	Prototype	*N*	Mean	Median	Standard deviation	Minimum	Maximum	*P* value	Result
**Tensile strength (N)**	S	5	2195.38	2182.20	581.81	1371.80	2913.70	**0.021**	**Statistically significant (S stronger)**
	C	5	1273.38	1315.00	282.72	974.40	1674.00		
	**Total**	10	1734.38	1522.90	649.70	974.40	2913.70		
**Maximum deflection (mm)**	S	5	32.27	33.30	4.16	25.74	37.20	**0.01**	
	C	5	14.18	14.04	5.76	6.21	21.44		
	**Total**	10	23.22	23.59	10.65	6.21	37.20		

*S* circumferential casting with a splint group, *C* circumferential casting alone group, *N* number of prototypes tested

## Discussion

The main finding in our study was that applying a splint before circumferential bandage makes the cast more resistant to bending loads, compared to a circumferential cast of the same weight (with the same quantity of material).

This fact allows us to get a more stable and resistant immobilization in conservative treatment of distal radius fractures, with the same quantity of plaster. Furthermore, the use of a splint (S group) allows a better adaptation to the anatomy of the forearm (authors’ own opinion). Moreover, this addition does not imply either a technical difficulty or higher costs.

No other study comparing the strength of different circular plaster casts configurations for distal radius fracture immobilization has been found, but some studies have compared different configurations of splints to get more resistance to bending forces.

Stewart et al. [16] compared different configurations of splinting and found that mounting reinforcement ridges on the splint could augment 100% the bending strength of the plaster with only a 20% increase in weight. This article emphasizes the great change in bending strength that can be achieved with just small changes in configuration, as used in our study. In fact, applying a splint under a circular cast makes similar to adding ridges to a splint, increasing bending resistance, similar to the study of Stewart et al. [16]. Theopold et al. [17] obtained similar results in a study quite similar to that of Stewart et al. [16], applying reinforcements to splints. Ridges or reinforcements make the cast more bulky, being sometimes disappointing to the patient. For our study, just adding a splint before applying the circumferential cast make no difference to the external aspect of the plaster (Figs. 1 and 2), being well tolerated by the patient.

The short plaster cast or splint is the most commonly used form of immobilization of distal radius fractures nowadays. Some studies [15, 20] showed that short plaster casts provide at least similar results to the long ones. We have used for this study a model of short plaster cast. Nevertheless, we consider that the results of this article for the wrist are also applicable to long plaster casts, as mechanical forces were applied distally to the elbow.

The main forces that should resist the cast are at the wrist. In a practical setting, usual forces and breaking of plaster occur at the wrist, so this is the “weak point” of the cast [20–22]. Moreover, the volar side of the wrist is considered, in classical teaching [19], the “weak point” because this area is more prone to hits and rubs that can weaken it. This is the reason that we wondered about the best way to avoid this form of breaking without increasing the weight or thickness of the cast. Moreover, mechanical testing was performed in this way, in flexion, to get the information about the “weak point” of the plaster, resembling usual forces. Mechanical testing was not performed in other directions because it is not the common way of failure of these casts. One limitation of the study may be found in the small number of prototypes tested, but we do not feel this as a problem because, despite the short series studied, we found statistically significant differences between S and C groups, so we have conclusive results and we can draw strong conclusions. Another limitation is that this is a mechanical basic study, so further clinical research is required. Other forms of applying circumferential plaster could be possible, but they are not so commonly used in Europe as the prototypes here studied. Applying the splint to the volar side of the wrist is a possibility, but in the practical setting is somehow difficult to apply and we do not have any report of putting the plaster in that way. So we did not feel it useful to study other plaster configurations.

The application of the results of this article may be widespread. As known, distal radius fractures involve 16% of the whole body’s fractures, and 90% of them are extraarticular [1]. As several authors refer [2–6], conservative treatment with plaster cast is the most used type of treatment in older patients, so the addition of a splint (S group) to the circular cast may be widely used in the daily routine. Moreover, distal radius fractures are even more frequent in childhood (physeal injuries), and the conservative treatment is also the most commonly type of treatment used [20–22].

## Conclusion

In conclusion, the application of a dorsal splint before the circular plaster cast for conservative treatment in distal radius fractures provide more strength to flexion forces than circular plaster cast performed with the same quantity of plaster. The strength is higher, with same weight and cost. We strongly recommend this form of application of plaster.

## Data Availability

The datasets used and/or analyzed during the current study are available from the corresponding author on reasonable request.

## Abbreviations

H0: Null hypothesis
H1: Alternative hypothesis
